# Context-specific drivers of non-regular long-lasting insecticidal net use in the Greater Mekong Subregion

**DOI:** 10.1186/s12936-026-05812-4

**Published:** 2026-01-31

**Authors:** Pyae Linn Aung, Piyarat Sripoorote, Myat Thu Soe, Pattamaporn Petchvijit, Poh Poh Aung, Khaing Zin Zin Htwe, Saranath Lawpoolsri, Jaranit Kaewkungwal, Liwang Cui, Daniel M. Parker, Myat Phone Kyaw, Jetsumon Sattabongkot

**Affiliations:** 1 Mahidol Vivax Research Unit, Faculty of Tropical Medicine, Mahidol University, Bangkok, Thailand; 2 Myanmar Health Network Organization, Yangon, Myanmar; 3 Department of Tropical Hygiene, Faculty of Tropical Medicine, Mahidol University, Bangkok, Thailand; 4 Division of Infectious Diseases and International Medicine, Department of Internal Medicine, Morsani College of Medicine, University of South Florida, 3720 Spectrum Boulevard, Suite 304, Tampa, FL 33612 USA; 5 Department of Population Health and Disease Prevention, Department of Epidemiology & Biostatistics, University of California, Irvine, CA USA

**Keywords:** Malaria, LLIN, GMS, Thailand, Myanmar, Risk factors

## Abstract

**Background:**

Long-lasting insecticidal nets (LLINs) are essential for malaria prevention, yet consistent use remains suboptimal. This study assessed LLIN use and associated factors in Thailand and Myanmar across diverse transmission contexts.

**Methods:**

We analyzed cross-sectional data from 13,459 individuals attending malaria service points in three districts in Thailand and two townships in Myanmar (2017–2024). LLIN use was categorized as daily, intermittent, or non-use. Logistic regression identified factors linked to non-regular use in each country. A directed acyclic graph (DAG) illustrated hypothesized causal pathways.

**Results:**

Among 3,062 participants in Myanmar, 16.9% reported intermittent or non-use. Higher odds were observed among individuals aged 5–14 years (aOR = 1.87, 95% CI: 1.29–2.75), 15–34 years (aOR = 3.42, 95% CI: 2.07–5.67), and ≥ 35 years (aOR = 4.42, 95% CI: 2.50–7.86), Rakhine ethnicity (aOR = 3.54, 95% CI: 2.76–4.57), residence in Paletwa (aOR = 20.9, 95% CI: 5.29–109), uncertain malaria history (aOR = 8.03, 95% CI: 3.61–18.4), and *Plasmodium falciparum* infection (aOR = 2.87, 95% CI: 2.02–4.06). Among 10,397 participants in Thailand, 31.2% reported intermittent or non-use. Significant factors included older age (aOR = 2.73, 95% CI: 2.07–3.62 for 15–34 years), male sex (aOR = 1.73, 95% CI: 1.56–1.91), agricultural occupation (aOR = 1.42, 95% CI: 1.04–1.95), residence in Bannang Sata (aOR = 17.9, 95% CI: 14.4–22.4) or Saba Yoi (aOR = 34.4, 95% CI: 23.3–52.3), *P. falciparum* (aOR = 3.61, 95% CI: 1.71–7.78), *P. vivax* (aOR = 2.74, 95% CI: 2.38–3.17), and lower odds with uncertain malaria history (aOR = 0.49, 95% CI: 0.35–0.68).

**Conclusion:**

Non-regular LLIN use was common and linked to demographic, occupational, and clinical factors. Context-specific strategies are needed to improve adherence and support malaria elimination goals.

## Background

The Greater Mekong Subregion (GMS), comprising six countries along the Mekong River, aims to achieve malaria elimination by 2030 [1]. China reached this goal in 2021, and several others have reported substantial progress [1, 2]. In 2023, Cambodia, Viet Nam, and the Lao People’s Democratic Republic recorded 1,384, 453, and 695 cases, representing reductions of 86.1%, 68.2%, and 80.2% from 2020, respectively [3]. In stark contrast, Myanmar reported 228,567 cases in 2023, about 3.8 times higher than in 2020, following the 2021 military coup that disrupted health services and displaced large populations into neighboring countries [3–5]. Thailand has since experienced a resurgence, with 16,684 cases in 2023, more than four times the 2020 figure; nearly 44% were imported from Myanmar [3, 6]. Sustained importation threatens elimination efforts, highlighting the need for effective vector control and strengthened prevention strategies in both Myanmar and border areas.

Malaria transmission is sustained through an epidemiological triad comprising the vector, the human host, and the parasite. Breaking this cycle requires preventing contact between infectious humans and competent *Anopheles* mosquitoes, or between infected mosquitoes and susceptible humans. In the GMS and elsewhere, adult vector control remains the cornerstone of malaria prevention, as larval source management is generally impractical for malaria vectors due to their dispersed and transient breeding habitats. The two primary strategies are the use of insecticide-treated nets, particularly long-lasting insecticidal nets (LLINs), and indoor residual spraying (IRS) [7]. LLINs, pre-treated with pyrethroid-based insecticides during manufacture, have been shown to reduce malaria incidence and vector survival in multiple settings, making them a first-line intervention recommended by World Health Organization (WHO) [8].

In the GMS, LLINs are distributed through national malaria control programs, typically on a two-year replacement cycle, with annual top-ups targeting populations at higher risk such as migrants, pregnant women, and young children. In line with WHO recommendations, distribution strategies in countries including Thailand and Myanmar aim to achieve coverage of approximately one LLIN for every two persons per household. However, despite these policies, actual household-level availability and utilization may vary due to factors such as population mobility, household sleeping arrangements, and timing between distribution rounds. Consequently, LLIN use remains suboptimal among certain groups, including working-age adults, forest-goers, and migrants [9–13]. Reported reasons for non-use include occupational constraints in environments where LLIN deployment is impractical, sociodemographic factors such as older age, low economic status, and perceptions related to discomfort in hot weather or concerns about insecticide-related reactions [9, 10, 13–15]. In response to increasing pyrethroid resistance among malaria vectors, some countries have begun introducing next-generation LLINs treated with dual active ingredients, although coverage remains limited [16–18]. IRS, which involves applying residual insecticides to indoor surfaces, is another effective intervention that can rapidly reduce vector populations and interrupt transmission. In Myanmar, IRS is generally implemented reactively during focal outbreaks, whereas in Thailand it is deployed as part of the “1–3-7” surveillance strategy to interrupt local transmission from identified index cases [19, 20]. Nevertheless, IRS is labor-intensive, requires high levels of community acceptance, and its effectiveness is constrained by insecticide resistance and limited operational coverage [21, 22].

People residing in areas of ongoing malaria transmission remain at continuous risk, particularly where efficient vector species such as *Anopheles minimus* and *An. dirus* are present, as documented in both Thailand and Myanmar [23, 24]. Even a single bite from an infectious mosquito can transmit malaria, making consistent personal protection critical [25]. While LLIN distribution coverage in the GMS is generally high, actual usage patterns vary. In some settings, individuals prefer conventional untreated nets due to privacy concerns (e.g. thicker or less transparent fabric) or discomfort in hot weather (e.g. larger mesh allowing more airflow and absence of insecticide-related odor or skin irritation) [10, 26, 27]. Furthermore, among those who report LLIN use, it is often unclear whether use is consistent every night or intermittent. These uncertainties highlight a critical evidence gap, particularly in the context of elimination goals, where sustaining high LLIN use is essential to prevent re-establishment of transmission. This study therefore aimed to assess everyday LLIN use and identify determinants of non-regular use in two malaria-endemic countries of the GMS, Thailand and Myanmar.

## Methods

### Study design and settings

This study employed a cross-sectional analysis of routinely collected individual-level data from people seeking malaria diagnosis or treatment at community-based malaria service points in two countries in the GMS between late 2017 and 2024.

**Fig. 1:**
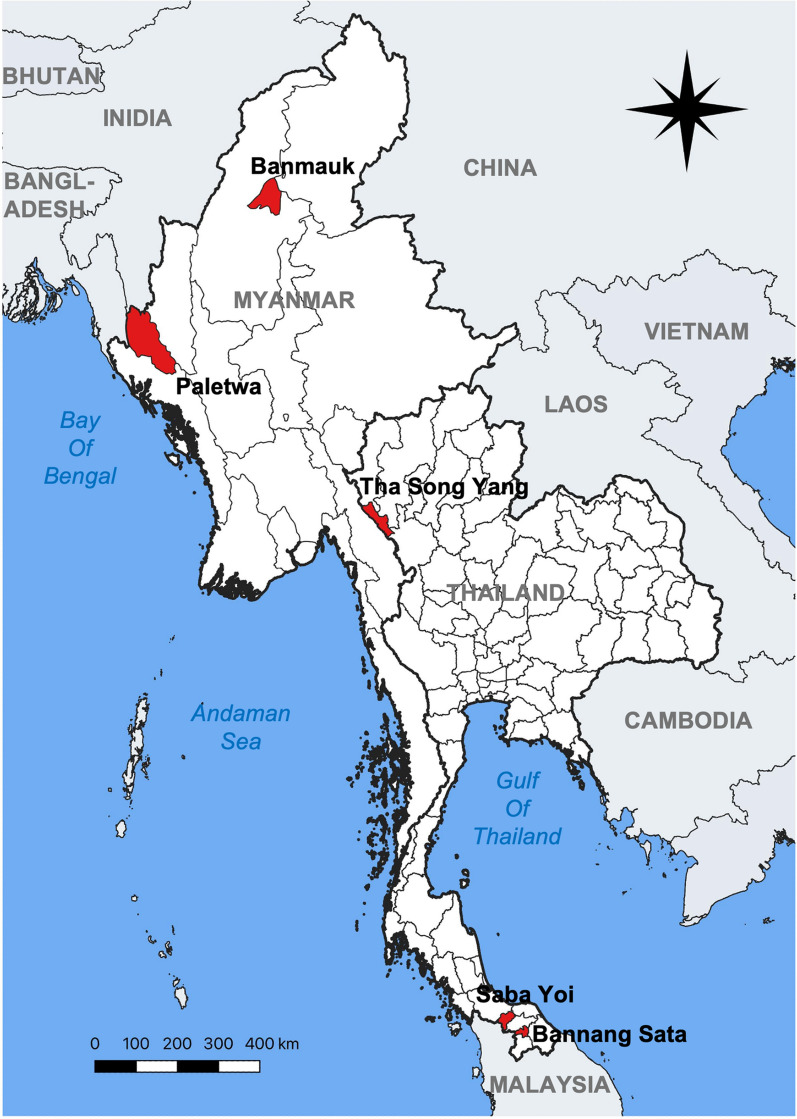
Geographic locations of the study sites in Thailand and Myanmar. The base map shapefiles were obtained from the DIVA-GIS database (https://diva-gis.org) and modified using QGIS for Mac (version 3.34.2-Prizren)

In Thailand, malaria transmission is predominantly concentrated in the western regions bordering Myanmar. For example, in 2024, Tak Province in western Thailand accounted for more than 48% of the country’s total reported malaria cases [6]. Among the nine districts in Tak Province, Tak Song Yang District was purposively selected due to its persistently high malaria endemicity. This district borders southeastern Myanmar, separated by the Moei River, which can be easily crossed on foot without passing through official checkpoints, particularly during the dry season. In contrast, southern provinces in Thailand report only residual malaria, with an emerging trend of *Plasmodium knowlesi* infections [28]. To represent varying endemicities and geographic contexts, two districts (Bannang Sata and Saba Yoi) with the highest malaria burden in the south, bordering Malaysia, were purposively selected. In total, three districts in Thailand were included in the study (Fig. 1). The selected villages in these districts are predominantly rural Karen communities along the Thai–Myanmar border, with many residents being migrants or refugees from Myanmar who have resided in Thailand for extended periods.

In each selected district, malaria services at the community level are delivered by 5–8 government-established malaria clinics (MCs). From each district, 2–3 MCs were selected based on recommendations from Provincial Public Health Officials and the feasibility of travel and logistics. The MCs provide malaria diagnosis and treatment free of charge to all individuals presenting with suspected symptoms, regardless of nationality, refugee status, or legal status (documented or undocumented), following a passive case detection (PCD) approach [29]. Malaria diagnosis is primarily performed using microscopy, with rapid diagnostic tests (RDTs) used in febrile patients for immediate diagnosis. All individuals of any age presenting to the selected MCs with fever or suspected malaria during the study period were eligible for inclusion.

In Myanmar, approximately 12 of the 330 townships, primarily located in Kayin, Chin, and Sagaing States, contributed nearly 70% of total reported malaria cases in recent years. Accordingly, Banmauk Township in Sagaing and Paletwa Township in Chin State were purposively selected as study sites. Banmauk is located in upper Myanmar, while Paletwa lies in the western region, bordering India and Bangladesh (Fig. 1). Based on recommendations from local health authorities and administrative leaders, 8–10 villages with high malaria incidence were selected in each township. In each village, at least one community-based village health volunteer (VHV) was assigned by the Vector Borne Disease Control (VBDC) team to provide malaria diagnostic and treatment services. These VHVs completed an initial 3–5-day training followed by annual refresher courses. Community members could access free malaria services from trained VHVs under the PCD approach [30, 31]. Malaria diagnosis was performed using combo RDTs detecting both *P. falciparum* and *P. vivax*. All community members who presented to the VHVs with fever or suspected malaria during the study period were eligible to participate.

### Data collection

MC staff and VHVs at the study sites collected demographic information using routine surveillance record forms. In addition, for this study, a standardized case record form (CRF) was developed in English and reviewed by malaria experts from both Thailand and Myanmar for content validity. The CRF was subsequently translated into Thai and Burmese using a back-translation method to ensure accuracy. The CRF collected individual-level sociodemographic information including age, sex, ethnicity, occupation, education, previous malaria experience, and malaria diagnosis results. It also included a section on the use of LLINs, categorized into three groups: used every day, used on some days of the week, or not used at all. Information on LLIN ownership or access was not collected; consequently, the “not used at all” category may include both individuals who did not use an available LLIN and those who lacked access to an LLIN.

Selected MC staff and VHVs received at least one day of field-based training, which covered Good Clinical Practice (GCP) principles, detailed instructions on completing the CRF, data collection procedures, informed consent processes, and data transmission protocols. The training included practical sessions, such as mock interviews where trainees role-played as service providers and patients.

While participants were waiting for their malaria diagnosis results, a trained data focal person administered the CRF through a brief face-to-face interview. Completing the entire CRF typically took 10–15 min. Once completed, the forms were scanned and uploaded to a secure, cloud-based data management system hosted at the Faculty of Tropical Medicine, Mahidol University. A dedicated data focal person at each study site oversaw this process. The central data management team regularly checked for errors or inconsistencies and resolved any queries through close communication with field teams.

### Data handling and analysis

Anonymized datasets covering the period from late 2017 to the end of 2024 were retrieved from the centralized data management system. Due to the impact of the COVID-19 pandemic and ongoing political instability in Myanmar, the Myanmar dataset included records from December 2017 to June 2021. In contrast, the Thailand dataset spanned from January 2018 to December 2024.

All individuals included in the datasets, regardless of age, sex, or other demographic characteristics, were considered eligible for inclusion. Data were reviewed for completeness and internal consistency. Records with unresolved queries or missing responses for key variables, such as LLIN use, were excluded. Additionally, individuals who reported using only conventional (non-insecticidal) nets were excluded, as the focus of this study was on the use of LLINs. In cases of repeated entries for a single individual within the same month, often due to follow-up visits, only the first record was retained to avoid overrepresentation. After cleaning, a total of 3,062 records from Myanmar and 10,397 from Thailand (13,459 total) were included in the final analysis. Because participants were recruited exclusively at malaria service points, the study population represents individuals who were seeking care for suspected malaria rather than a random sample of the general community in each setting.

The primary outcome of interest was the pattern of LLIN use, based on participant responses regarding bed net use during the week preceding the interview. Responses were categorized into three groups: (1) used every day, (2) used on some days, and (3) did not use at all. For analytical purposes, a binary variable was created by combining "some days" and "not at all" into a single category, with "everyday use" coded as the reference group, in alignment with malaria prevention recommendations.

Descriptive statistics (frequencies and percentages) were then used to summarize participant characteristics and LLIN use. The proportions of LLIN use categories, along with their 95% confidence intervals (CIs), were presented using bar charts. The chi-squared test was used to assess differences in LLIN use between Thailand and Myanmar. To explore factors associated with non-regular LLIN use, separate univariate and multivariable logistic regression models were applied for each country due to differences in socioeconomic conditions, geographic settings, and malaria control strategies. All variables from the univariate analyses were included in the multivariable models regardless of statistical significance, to ensure that all potential confounders were considered. Adjusted odds ratios (aORs) with 95% CIs from both countries were then compiled and visualized in a single random forest plot to facilitate cross-country comparison of effect sizes. All variance inflation factor (VIF) values were below 1.7, indicating no significant multicollinearity. All analyses were performed using RStudio (version 2025.05.0 + 496; Posit, PBC).

A directed acyclic graph (DAG) was constructed to illustrate hypothesized causal relationships between demographic and contextual variables and non-regular LLIN use [32]. Variable selection and pathway justification were informed by prior knowledge and supported by Cramér’s V correlation analysis, which identified weak to moderate associations (Cramér’s V < 0.6) among most predictors, except for a strong relationship between ethnicity and study site in the Myanmar dataset (Cramér’s V = 0.98). To ensure comparability across contexts, only variables with relevance and comparable associations in both countries were retained in the DAG. Factors found to be relevant in one setting but not the other were excluded from the final model to maintain cross-country balance and generalizability of the hypothesized pathways.

## Results

### Sociodemographic and malaria-related characteristics of participants by country

Among the 3,062 participants from Myanmar, the majority were aged 15 years or older (62.5%) and male (57.1%). Most participants identified as Chin (41.3%) or Shan (39.0%), and nearly half were engaged in agriculture (48.4%), followed by students or children (41.6%). Over half (55.7%) had completed primary school, while 17.5% reported no formal education. Only 4.1% reported a history of malaria in the past 12 months. Malaria diagnoses by RDTs revealed *P. falciparum* and *P. vivax* infections in 5.8% and 5.4% of participants, respectively (Table 1).

**Table 1:** Demographic, occupational, and malaria-related characteristics of study participants in Myanmar and Thailand (n = 13,459)

Characteristic	Myanmar (n = 3062)	Thailand (n = 10,397)
	n (%)	n (%)
Age group (years)		
< 5	388 (12.7)	1057 (10.2)
5–14	762 (24.9)	2981 (28.7)
15–34	962 (31.4)	3189 (30.6)
≥ 35	950 (31.0)	3170 (30.5)
Sex		
Female	1314 (42.9)	4313 (41.5)
Male	1748 (57.1)	6084 (58.5)
Ethnicity		
Chin	1264 (41.3)	–
Rakhine	528 (17.2)	–
Shan	1195 (39.0)	–
Other^a^	75 (2.5)	68 (0.6)
Karen	–	7317 (70.4)
Thai	–	3012 (29.0)
Occupation		
Agriculture/Farmer	1481 (48.4)	3459 (33.3)
Child/Student	1275 (41.6)	4433 (42.6)
Unemployed	87 (2.8)	335 (3.2)
Other^b^	219 (7.2)	2170 (20.9)
Malaria in past 12 months		
Yes	127 (4.1)	457 (4.4)
No	2878 (94.0)	9514 (91.5)
Do not remember	57 (1.9)	426 (4.1)
Study site		
Banmauk	1236 (40.4)	–
Paletwa	1826 (59.6)	–
Bannang Sata	–	878 (8.5)
Saba Yoi	–	294 (2.8)
Tha Song Yang	–	9225 (88.7)
Malaria diagnosis^c^		
Negative	2708 (88.4)	9326 (89.7)
* P. falciparum*	177 (5.8)	30 (0.3)
* P. vivax*	165 (5.4)	1023 (9.8)
Other^d^	12 (0.4)	18 (0.2)

^a^“Ethnicity (Other)” includes: Myanmar—Mon, Burmese, Karen, Kadu, Kachin, etc.; Thailand—Mon, Chin, Chinese, Kachin, Khmer, Myanmar, Rakhine, etc.

^b^“Occupation (Other)” includes: Myanmar—daily wages, labor, livestock, lumberjack, merchant, monk, etc.; Thailand—government officer, labor, livestock, merchant, monk, soldier/police, etc

^c^Malaria diagnosis was conducted using RDTs in Myanmar and microscopy in Thailand

^d^“Diagnosis (Other)” includes: Myanmar—*P. falciparum* + *P. vivax*; Thailand—*P. falciparum* + *P. vivax*, *P. knowlesi*, *P. ovale*, *P. malariae*

Of the 10,397 participants in Thailand, most were aged 15 years or older (61.1%) and male (58.5%). The majority were of Karen ethnicity (70.4%), and the most common occupations were student/child (42.6%) and agriculture (33.3%). Nearly 40% had no formal education, while 32.7% had completed primary-level education. Malaria history in the past year was reported by 4.4% of participants. Microscopy-based diagnosis revealed a high prevalence of *P. vivax* (9.8%) and a low detection rate of *P. falciparum* (0.3%) (Table 1).

### LLIN use among participants

**Fig. 2:**
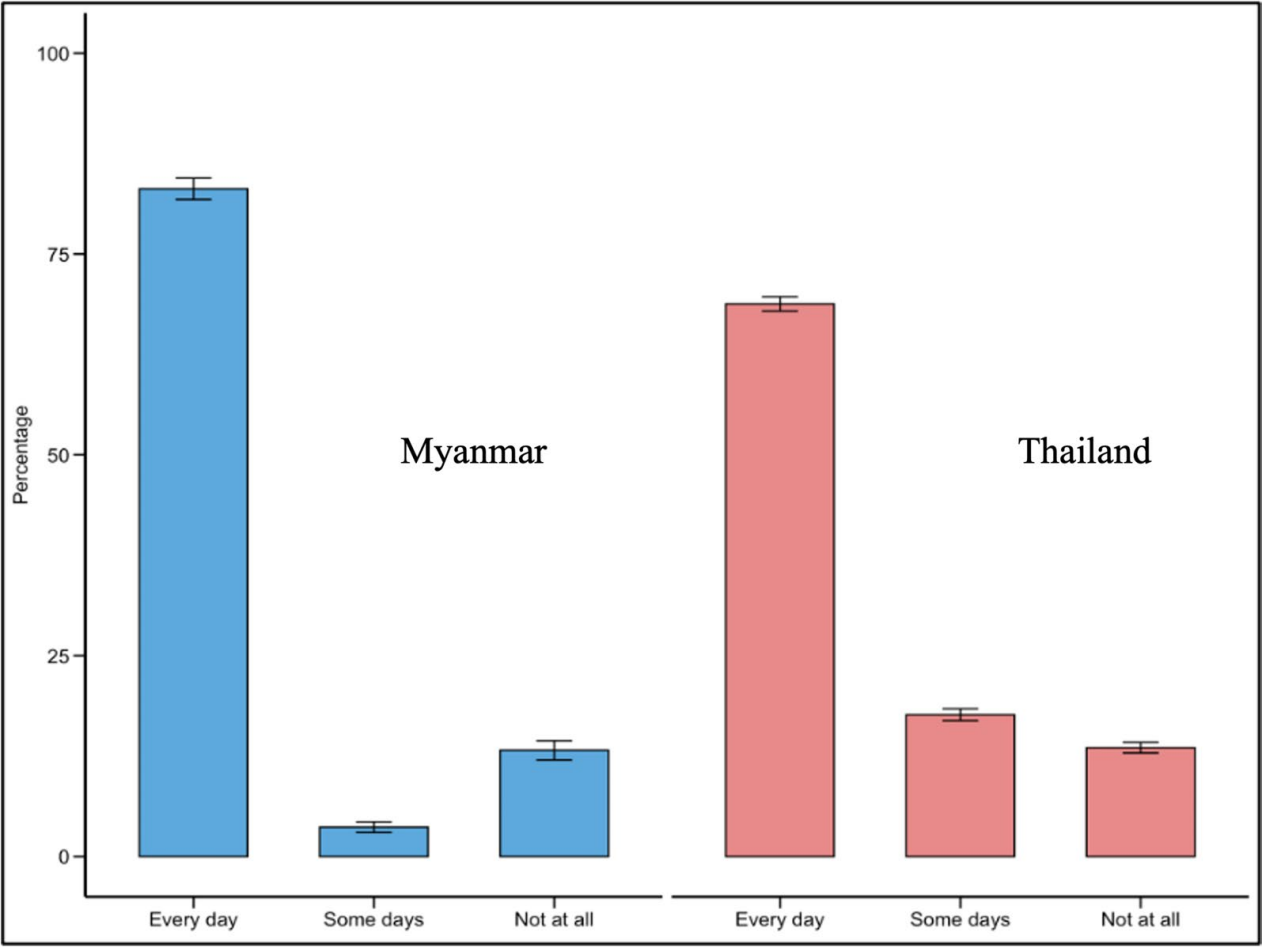
Use of LLINs among participants in Myanmar and Thailand

Self-reported LLIN use was high overall but differed significantly between countries (*p* < 0.001). In Myanmar, 83.1% (95% CI: 81.8–84.4%) of respondents reported using LLINs every day, compared to 68.8% (95% CI: 67.9–69.7%) in Thailand. Intermittent use ('some days') was reported by 3.7% (95% CI: 3.0–4.3%) of participants in Myanmar and 17.7% (95% CI: 16.9–18.4%) in Thailand. Non-use of LLINs (which may reflect either lack of access to an LLIN or non-use despite access) was reported by 13.2% (95% CI: 12.0–14.4%) and 13.6% (95% CI: 12.9–14.2%) of respondents in Myanmar and Thailand, respectively (Fig. 2**)**.

### Distribution of LLIN intermittent or non-use by participant characteristics

Table 2 summarizes LLIN use patterns in Myanmar. Intermittent or non-use was reported by 17–18% of participants aged 5 years or older, compared to only 13.1% among children under 5. The highest proportions of non-use were observed among individuals of Rakhine ethnicity (43.4%), residents of Paletwa (26.1%), and participants with *P. falciparum* infection (44.1%). Non-use was also frequent among those who could not recall their malaria history (59.6%) and among participants in “other” occupations such as merchants or laborers (31.1%), while differences between males and females were small (17.2% vs. 16.5%).

**Table 2:** Patterns of intermittent or non-use of LLINs among participants in Myanmar (n = 3062)

Characteristic	LLIN		cOR (95%CI)
	Everyday use (n = 2545)	Intermittent/Non-use (n = 517)	
	n (%)	n (%)	
Age group (years)			
< 5	337 (86.9)	51 (13.1)	Ref
5–14	626 (82.2)	136 (17.8)	1.44 (1.02–2.05)
15–34	795 (82.6)	167 (17.4)	1.39 (1.00–1.96)
≥ 35	787 (82.8)	163 (17.2)	1.37 (0.98–1.94)
Sex			
Female	1097 (83.5)	217 (16.5)	Ref
Male	1448 (82.8)	300 (17.2)	1.05 (0.87–1.27)
Ethnicity			
Chin	1031 (81.6)	233 (18.4)	Ref
Rakhine	299 (56.6)	229 (43.4)	3.39 (2.71–4.24)
Shan	1156 (96.7)	39 (3.3)	0.15 (0.10–0.21)
Other^a^	59 (78.7)	16 (21.3)	1.20 (0.66–2.07)
Occupation			
Unemployed	68 (78.2)	19 (21.8)	Ref
Agriculture/Farmer	1280 (86.4)	201 (13.6)	0.56 (0.34–0.98)
Child/Student	1046 (82.0)	229 (18.0)	0.78 (0.47–1.36)
Other^b^	151 (68.9)	68 (31.1)	1.61 (0.91–2.95)
Malaria in past 12 months			
Yes	98 (77.2)	29 (22.8)	Ref
No	2424 (84.2)	454 (15.8)	0.63 (0.42–0.99)
Do not remember	23 (40.4)	34 (59.6)	5.00 (2.58–9.92)
Study site			
Banmauk	1196 (96.8)	40 (3.2)	Ref
Paletwa	1349 (73.9)	477 (26.1)	10.6 (7.69–15.0)
Malaria diagnosis^c^			
Negative	2313 (85.4)	395 (14.6)	Ref
* P. falciparum*	99 (55.9)	78 (44.1)	4.61 (3.36–6.32)
* P. vivax*	124 (75.1)	41 (24.9)	1.94 (1.32–2.78)
Other^d^	9 (75.0)	3 (25.0)	1.95 (0.43–6.57)

^a^“Ethnicity (Other)” includes Mon, Burmese, Karen, Kadu, Kachin, etc

^b^“Occupation (Other)” includes daily wages, labor, livestock, lumberjack, merchant, monk, etc

^c^Malaria diagnosis was conducted using RDTs

^d^“Diagnosis (Other)” includes *P. falciparum* + *P. vivax*.; cOR: Crude odds ratio; 95% CI: 95% Confidence interval

In Thailand, intermittent or non-use of LLINs was reported by 23–40% of participants aged 5 years or older, compared to only 11.6% among children under 5 (Table 3). The highest proportions of non-use were observed among residents of Saba Yoi (89.8%) and Bannang Sata (81.2%), followed by participants with *P. falciparum* (56.7%) or *P. vivax* infection (47.3%). Non-use was also common among those in agricultural (40.4%) and other occupations such as merchants, soldiers, or laborers (38.8%), and among individuals of Thai ethnicity (46.8%) compared to Karen (24.7%). Males reported higher non-use than females (35.5% vs. 25.1%).

**Table 3:** Patterns of intermittent or non-use of LLINs among participants in Thailand (n = 10,397)

Characteristic	LLIN		cOR (95%CI)
	Everyday use (n = 7152)	Intermittent/Non-use (n = 3245)	
	n (%)	n (%)	
Age group (years)			
< 5	934 (88.4)	123 (11.6)	Ref
5–14	2300 (77.2)	681 (22.8)	2.25 (1.84–2.77)
15–34	1920 (60.2)	1269 (39.8)	5.02 (4.12–6.16)
≥ 35	1998 (63.0)	1172 (37.0)	4.45 (3.66–5.47)
Sex			
Female	3229 (74.9)	1084 (25.1)	Ref
Male	3923 (64.5)	2161 (35.5)	1.64 (1.51–1.79)
Ethnicity			
Karen	5508 (75.3)	1809 (24.7)	Ref
Thai	1602 (53.2)	1410 (46.8)	2.68 (2.45–2.93)
Other	42 (61.8)	26 (38.2)	1.88 (1.14–3.06)
Occupation			
Unemployed	274 (81.8)	61 (18.2)	Ref
Agriculture/Farmer	2061 (59.6)	1398 (40.4)	3.05 (2.31–4.09)
Child/Student	3490 (78.7)	943 (21.3)	1.21 (0.92–1.63)
Other	1327 (61.2)	843 (38.8)	2.85 (2.15–3.85)
Malaria in past 12 months			
Yes	291 (63.7)	166 (36.3)	Ref
No	6564 (69.0)	2950 (31.0)	0.79 (0.65–0.96)
Do not remember	297 (69.7)	129 (30.3)	0.76 (0.57–1.01)
Study site			
Tha Song Yang	6957 (75.4)	2268 (24.6)	Ref
Bannang Sata	165 (18.8)	713 (81.2)	13.3 (11.1–15.8)
Saba Yoi	30 (10.2)	264 (89.8)	27.0 (18.8–40.3)
Malaria diagnosis^a^			
Negative	6598 (70.7)	2728 (29.3)	Ref
* P. falciparum*	13 (43.3)	17 (56.7)	3.16 (1.54–6.65)
* P. vivax*	539 (52.7)	484 (47.3)	2.17 (1.91–2.47)
Other	2 (11.1)	16 (88.9)	19.3 (5.50–122)

^a^“Ethnicity (Other)” includes Mon, Chin, Chinese, Kachin, Khmer, Myanmar, Rakhine, etc

^b^“Occupation (Other)” includes government officer, labor, livestock, merchant, monk, soldier/police, etc

^c^Malaria diagnosis was conducted using microscopy

^d^“Diagnosis (Other)” includes *P. falciparum* + *P. vivax*, *P. knowlesi*, *P. ovale*, *P. malariae*.; cOR: Crude odds ratio; 95% CI: 95% Confidence interval

### Correlates of intermittent or non-use of LLINs among participants in Myanmar and Thailand

**Fig. 3:**
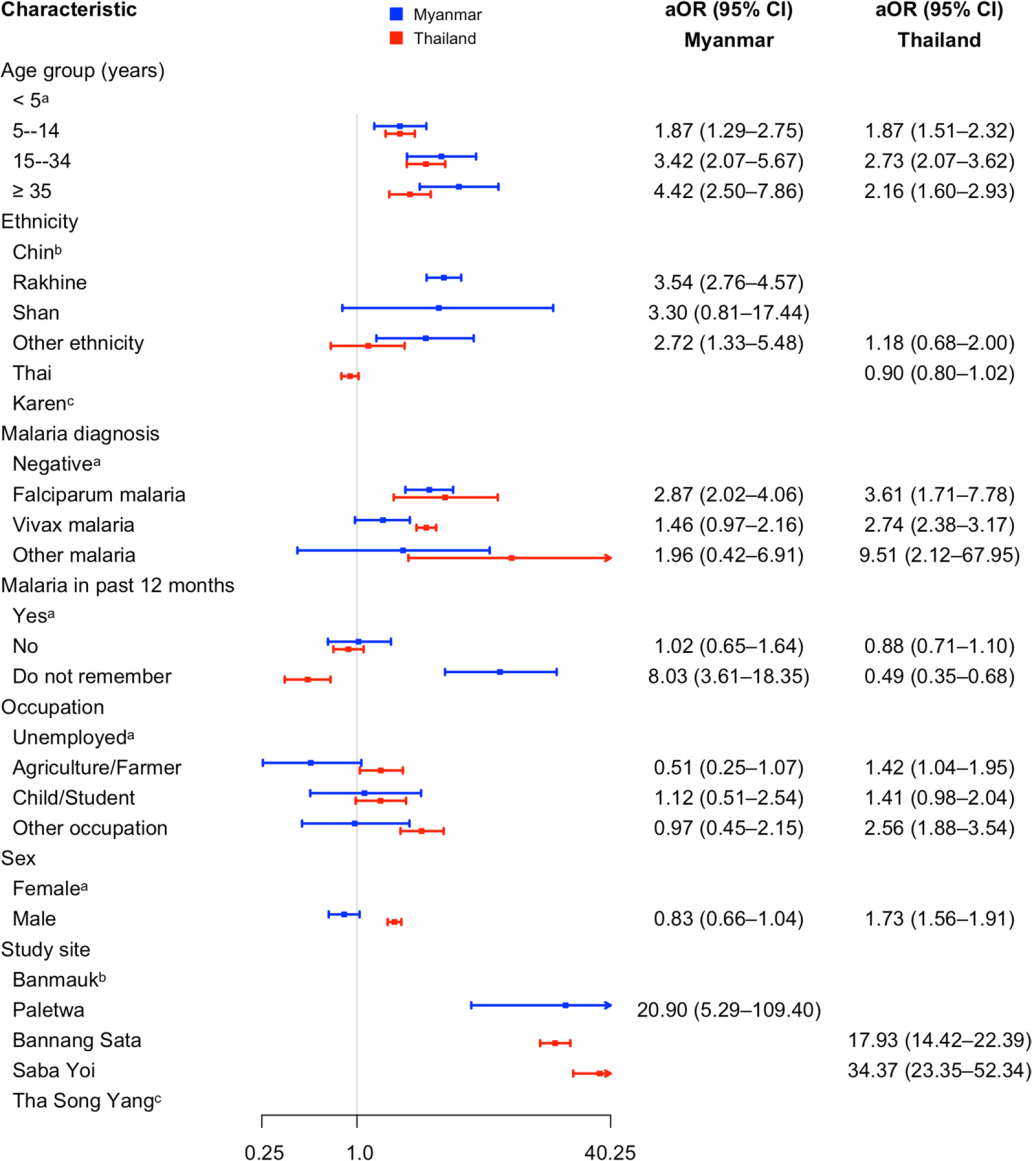
Adjusted associations with intermittent or non-use of LLINs in Myanmar and Thailand [^a^Reference category for both countries; ^b^Reference category for Myanmar; ^c^Reference category for Thailand]

In Myanmar, intermittent or non-use of LLINs was significantly associated with several demographic and clinical factors (Fig. 3). Compared to children under 5 years, older participants had progressively higher odds of non-daily use, including those aged 5–14 years (aOR = 1.87, 95% CI: 1.29–2.75), 15–34 years (aOR = 3.42, 95% CI: 2.07–5.67), and ≥ 35 years (aOR = 4.42, 95% CI: 2.50–7.86). Rakhine ethnicity was associated with substantially increased odds of intermittent or non-use (aOR = 3.54, 95% CI: 2.76–4.57), as was ethnicity categorized as “other” (aOR = 2.72, 95% CI: 1.33–5.48). Participants residing in Paletwa had significantly higher odds of non-use compared to Banmauk (aOR = 20.9, 95% CI: 5.29–109.40). Those who were unsure of their malaria history were more likely to report non-daily LLIN use (aOR = 8.03, 95% CI: 3.61–18.35), as were individuals diagnosed with *P. falciparum* infection (aOR = 2.87, 95% CI: 2.02–4.06).

In Thailand, several factors were significantly associated with intermittent or non-use of LLINs (Fig. 3). Compared to children under 5 years, participants aged 5–14 years had higher odds of non-daily use (aOR = 1.87, 95% CI: 1.51–2.32), with even greater odds among those aged 15–34 years (aOR = 2.73, 95% CI: 2.07–3.62) and those aged 35 years or older (aOR = 2.16, 95% CI: 1.60–2.93). Males were more likely than females to report non-regular LLIN use (aOR = 1.73, 95% CI: 1.56–1.91). Participants working in agriculture or farming had higher odds compared to the unemployed (aOR = 1.42, 95% CI: 1.04–1.95), and those in other occupations such as government officers, merchants, and laborers also had increased odds (aOR = 2.56, 95% CI: 1.88–3.54). Participants who did not recall their malaria history had significantly lower odds of non-use (aOR = 0.49, 95% CI: 0.35–0.68). Compared to Tha Song Yang, residents of Bannang Sata had substantially higher odds of non-use (aOR = 17.9, 95% CI: 14.42–22.39), as did those in Saba Yoi (aOR = 34.37, 95% CI: 23.35–52.34). Malaria infection was also associated with increased odds of intermittent or non-use, with *P. falciparum* (aOR = 3.61, 95% CI: 1.71–7.78), *P. vivax* (aOR = 2.74, 95% CI: 2.38–3.17), and other *Plasmodium* species (aOR = 9.51, 95% CI: 2.12–67.95), all showing significant associations.

### Predictors of non-regular LLIN use and hypothesized causal pathways

**Fig. 4:**
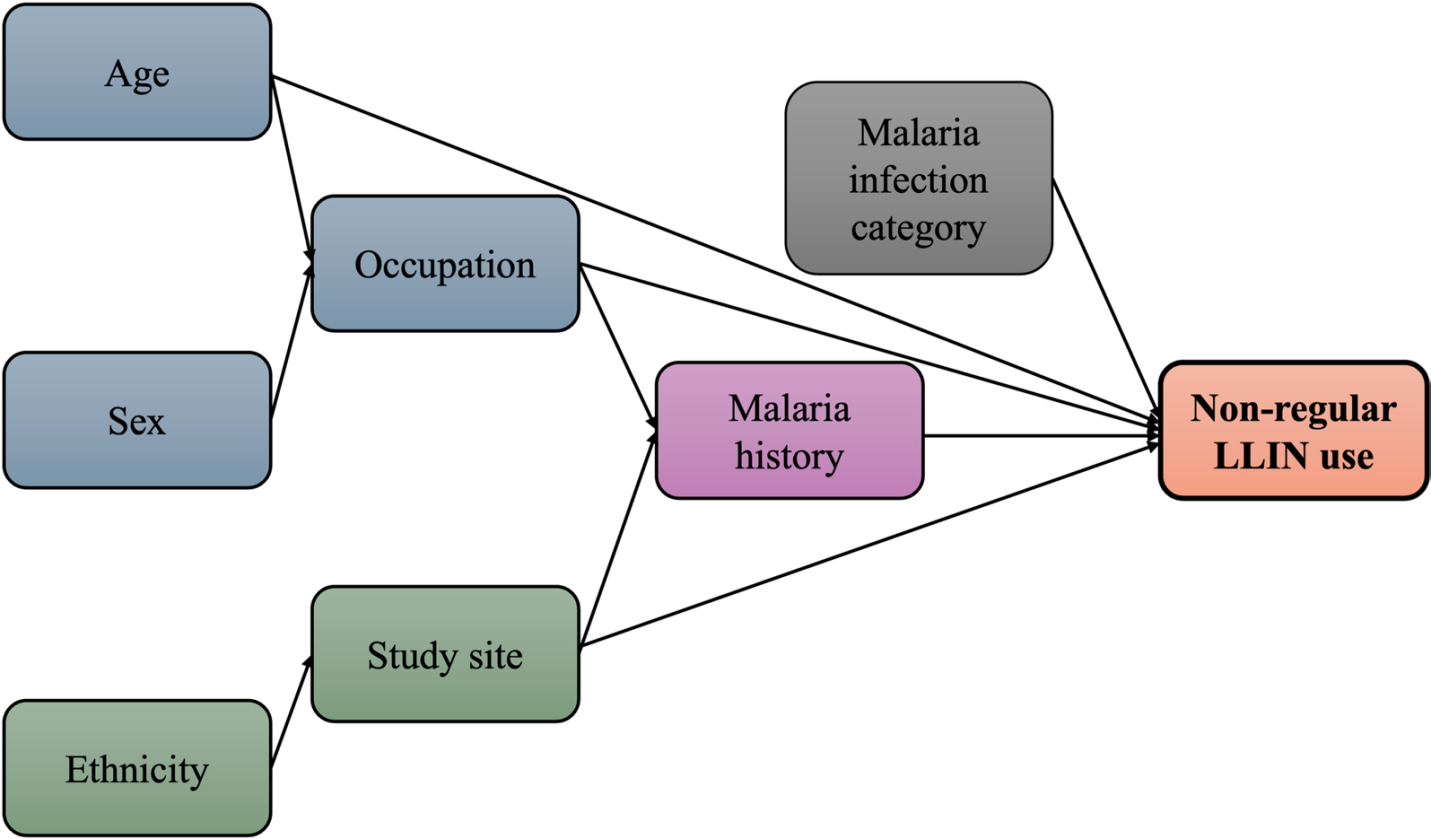
Directed acyclic graph (DAG) showing hypothesized predictors of non-regular LLIN use

Figure 4 presents a DAG illustrating the hypothesized relationships between demographic, contextual, and exposure-related factors associated with non-regular LLIN use. The DAG highlights the potential influence of age, sex, ethnicity, occupation, study site, and malaria history on LLIN use behavior. This framework supported the interpretation of observed associations and informed the multivariable analysis.

## Discussion

Although prevention remains a cornerstone of malaria control and elimination, particularly in interrupting onward transmission, consistent daily use of preventive measures such as LLINs remains suboptimal in many settings, even where routine distribution policies are in place. This is especially concerning in areas with ongoing transmission, where even a single infective mosquito bite can lead to infection [25]. Daily LLIN use during sleep is therefore essential. In our study, while a majority of participants reported using LLINs, a substantial proportion did not use them every day. Nevertheless, the proportion of participants in Myanmar who reported daily LLIN use was higher than in previous studies, which documented usage rates of approximately 15.3% in the general population [9], 18.4% among pregnant women [26], 19.5% among children under five [33], and 39.0–68.3% among mobile and migrant populations [11, 12, 27]. Similarly, the proportion observed in Thailand exceeded previous reports of 53.1% in northeastern provinces [10], 44.3% among migrants [13], and 8.6% among forest-related workers [10]. Multiple factors were found to be statistically associated with non-regular use in both countries, and the DAG helped contextualize these associations. However, since participants in this study were individuals seeking malaria diagnosis or treatment and who self-reported LLIN use, the prevalence may be overestimated. Nonetheless, in addition to ensuring widespread LLIN distribution, efforts must also prioritize promoting consistent daily use, particularly in areas with high malaria receptivity and vulnerability. Future studies employing qualitative methods are warranted to explore barriers to daily LLIN use in greater depth.

In both countries, individuals aged over five years were significantly less likely to report daily use of LLINs. This may reflect cultural practices in Southeast Asia, where young children, especially those perceived as more vulnerable, are often prioritized for the use of protective resources within households [9, 10, 34]. While current national guidelines in both countries recommend one LLIN for every two people, this standard may not always be practical, particularly in households where individuals prefer to sleep separately, resulting in insufficient coverage [3, 35]. Additionally, as indicated in the DAG, occupational exposures may contribute to lower LLIN use. For example, adult males in peripheral or forested areas may spend nights outdoors for work, where hanging and using LLINs is often impractical [10, 13, 36, 37]. In such contexts, alternative preventive measures, such as insect repellents, mosquito coils, or hammock nets, may be more appropriate. However, there is often a perception that malaria risk is confined to forested areas, and individuals residing or working in town centers may underestimate their vulnerability [9, 33, 38]. This may explain the lower LLIN use observed among government officers, monks, and soldiers, whose activities are typically based in urban or peri-urban settings. Promoting consistent LLIN use across all demographic and occupational groups, alongside improved messaging about malaria risk, regardless of setting, is critical for sustained prevention.

Although all study sites were categorized as active malaria foci, the level of endemicity varied widely between locations. For instance, Paletwa in western Myanmar reported an annual parasite incidence (API) of 15.8 per 1,000 population in 2018, whereas Banmauk had an API of only 3.8 per 1,000 in the same year [39, 40]. Similarly, in Thailand, Tha Song Yang District recorded over 2,000 malaria cases in 2024, compared to only a few cases in Bannang Sata and Saba Yoi, respectively [6]. Notably, participants from areas with lower malaria endemicity were less likely to report daily use of LLINs. This variation likely reflects the influence of local epidemiological conditions and perceptions of risk. Ideally, residents should be kept informed about the malaria situation in their areas through ongoing health education and community engagement by local healthcare providers. In line with the Health Belief Model, individuals in higher transmission areas may be more engaged in malaria-related interventions and, therefore, more likely to perceive the benefits of LLIN use [41]. However, it remains uncertain whether lower malaria incidence is the result of better preventive behaviors or whether reduced use of LLINs contributes to ongoing transmission. Malaria transmission is also influenced by multiple factors, including host-vector interactions, environmental conditions, and the availability and accessibility of health services [42–44]. Moreover, in some settings, health program funding is linked to disease burden, and reductions in funding following decreased incidence may inadvertently lead to resurgence [45]. As highlighted in the DAG, behavioral patterns also appear to align with the dominant ethnic groups in specific regions. For example, Paletwa is predominantly inhabited by the Rakhine ethnic group, who also exhibited significantly lower rates of daily LLIN use.

Numerous studies have demonstrated the effectiveness of LLINs in reducing malaria transmission by limiting human-vector contact [1, 46, 47]. Despite increasing concerns about insecticide resistance, LLINs remain a cornerstone of vector control strategies across endemic regions [3, 18]. In this study, individuals with confirmed malaria infections had significantly higher odds of reporting non-use or intermittent use of LLINs, reinforcing the protective role of consistent net use. This association has also been observed in other studies [48–51]. While large-scale LLIN distribution campaigns continue in both countries, including the provision of approximately 837,377 LLINs in Myanmar and 532,941 in Thailand in 2023, distribution alone is insufficient [3]. Programs must prioritize not only ensuring access but also promoting correct and regular use of LLINs, especially in areas approaching elimination status. This is particularly important given the emphasis on interrupting localized transmission through both prevention and timely treatment. A recent resurgence of malaria in northwestern Thailand, near the Myanmar border, underscores this concern. Reports attributed the increase in cases to potential importation of infections by Myanmar migrants, which subsequently contributed to transmission among local populations [6]. Importantly, the resurgence also highlighted gaps in preventive measures, including inconsistent LLIN use among both migrants and local residents.

Thailand and Myanmar differ substantially in terms of health system structure and malaria control capacity. Thailand generally benefits from greater resources and more formalized infrastructure for malaria prevention and treatment. In Thailand, malaria diagnosis and treatment are primarily delivered through trained healthcare personnel at dedicated malaria clinics [29]. In contrast, community-based services in Myanmar are largely provided by VHVs, who are responsible for diagnosing and treating malaria at the local level [30, 31]. The quality, scope, and consistency of services offered by VHVs may vary, potentially influencing community trust and engagement with malaria interventions.

Differences in socioeconomic status between the two countries may also shape health behaviors and perceptions. For instance, individuals in Myanmar may have limited exposure to formal health education, leading to uncertainty about previous malaria episodes or full reliance on advice from VHVs [31, 52, 53]. Conversely, in Thailand, where individuals have broad access to malaria services, there may be less urgency or perceived need for consistent preventive behavior, as care is readily accessible [54]. These dynamics may explain why, among participants who could not recall their prior malaria history, those from Myanmar were less likely to use LLINs daily, whereas those from Thailand reported higher LLIN adherence despite similar uncertainty.

This study has several notable strengths and limitations. It is one of the first to report detailed patterns of LLIN use across two malaria-endemic countries in the GMS, using a large dataset of over 13,000 records collected over a five-year period from multiple sites with varying levels of malaria transmission. The geographic breadth and temporal scope provide a robust foundation for understanding LLIN use in real-world settings. However, the reliance on self-reported data introduces potential biases, including recall and social desirability bias, which may affect the accuracy of reported behaviors. Additionally, while participants reported LLIN use, the study did not assess the condition of the nets (e.g., presence of holes or insecticidal efficacy) or usage practices such as frequency of washing, which may influence effectiveness [48]. The study also did not capture LLIN ownership, meaning that some participants who did not use a net daily may have lacked access to one, leading to underestimation of structural barriers. Another limitation is that the study population included only individuals who presented at malaria service points, which may not be representative of the broader community. One study site in Thailand is located along the border with Myanmar and included many participants originally from Myanmar, meaning the proportions of non-use or irregular use might be overestimated and not fully representative of LLIN use patterns within Thailand. Finally, data from Myanmar were collected before the onset of the political crisis in early 2021 and the associated disruption of health services and may not reflect current conditions following the combined impact of the COVID-19 pandemic and political instability.

## Conclusions

Despite widespread distribution of LLINs, consistent daily use remains suboptimal, particularly among older individuals, certain occupational groups, and populations residing in low-transmission areas. These findings underscore the need for context-sensitive strategies that go beyond net distribution to promote behavioral adherence, address structural barriers, and tailor health messaging. As countries in the region strive toward malaria elimination, sustaining high LLIN usage among at-risk populations remains a critical component of vector control and community protection.

## Data Availability

All data generated or analyzed during this study are included in the article. The anonymized raw dataset is available from the corresponding author upon reasonable request.

## Abbreviations

aOR: Adjusted odds ratio
CI: Confidence interval
cOR: Crude odds ratio
CRF: Case record form
DAG: Directed acyclic graph
GCP: Good clinical practice
GMS: Greater Mekong Subregion
LLIN: Long-lasting insecticidal net
MC: Malaria clinic
PCD: Passive case detection
RDT: Rapid diagnostic test
VBDC: Vector Borne Disease Control
VHV: Village health volunteer
VIF: Variance inflation factor
WHO: World Health Organization
